# EpCAM as Modulator of Tissue Plasticity

**DOI:** 10.3390/cells9092128

**Published:** 2020-09-19

**Authors:** François Fagotto

**Affiliations:** CRBM, University of Montpellier and CNRS, 34293 Montpellier, France; francois.fagotto@crbm.cnrs.fr

**Keywords:** Epithelial Cell Adhesion Molecule, Trop1, Trop2, TACSD, metastasis, cell–cell adhesion, cell–matrix adhesion, intercellular migration, actomyosin contractility, cortical tension

## Abstract

The Epithelial Cell Adhesion Molecule or EpCAM is a well-known marker highly expressed in carcinomas and showing a strong correlation with poor cancer prognosis. While its name relates to its proposed function as a cell adhesion molecule, EpCAM has been shown to have various signalling functions. In particular, it has been identified as an important positive regulator of cell adhesion and migration, playing an essential role in embryonic morphogenesis as well as intestinal homeostasis. This activity is not due to its putative adhesive function, but rather to its ability to repress myosin contractility by impinging on a PKC signalling cascade. This mechanism confers EpCAM the unique property of favouring tissue plasticity. I review here the currently available data, comment on possible connections with other properties of EpCAM, and discuss the potential significance in the context of cancer invasion.

## 1. Introduction

The Epithelial Cell Adhesion Molecule (EpCAM) is cell membrane protein originally identified as an antigen derived from tumours. In parallel, it was also identified through its enrichment in the placenta, thus acquired its alternate name Trop1 for Trophoblast cell surface antigen 1 [1]. In mammals, EpCAM is expressed in embryonic epithelia, but the levels usually drop as cells reach terminal differentiation [2,3]. Enhanced expression of EpCAM is associated with active proliferations of neoplastic or normal tissues (e.g., [4,5,6]). Work by Litvinov lab showed evidence for a function as a homophilic cell–cell adhesion molecule (CAM) [7,8,9,10,11], from which the name of EpCAM originates. EpCAM is unique to vertebrates, but highly conserved from fish to human. In amniotes, a second gene has appeared by retrotransposition, which is commonly called Trop2, and is conserved in all birds and mammals. Considering the close sequence similarity between EpCAM and Trop2 (48% identity and 78% similarity in humans), the high conservation of EpCAM in all vertebrates (and the conserved enrichment in epithelial tissues), while the trophectoderm only exists in mammals, a better name for Trop2 would be EpCAM2. To compromise between coherence and common terminology, I will use here the double name EpCAM2/Trop2. Note that Trop2 was also independently characterized as a molecule capable of modulating intracellular calcium, and was thus given the name of tumour-associated calcium signal transducer [12]; one also finds, in the literature and databases, the terms TACSTD1/2.

## 2. General Features of EpCAM

EpCAM and EpCAM2/Trop2 are single transmembrane glycoproteins, with a unique sequence and a unique 3D structure (reviewed in [13]). They are unrelated to any known CAM. The only detected similarity concerns a structural motif in the extracellular domain that has the cysteine organization of a thyroglobulin-type A1 domain. The extracellular domain is extremely compact, and sticks out only 5 nm outside of the membrane lipid bilayer, which is a rather short distance (for comparison, the cadherin extracellular domain is about 15 nm long). The protein is known to be glycosylated, at possibly up to three sites, although the exact role of these modification is unclear [14,15]. The transmembrane domain (TM) is also remarkably conserved and prominently rich in valine, while most TM domains tend to be enriched in leucine [16]. Although experimental evidence is still missing, this peculiar composition could be related to the property of EpCAM to associate with special lipid membrane domains, called TEMs, organized through a network of transmembrane proteins called tetraspanins [17,18]. The cytoplasmic *C*-terminal sequence is exceptionally short. It has only 26 amino acids in humans, and as little as 18 in fish. However, it includes highly conserved residues, which, as we will see, play a key role in EpCAM function. EpCAM has been shown to form stable lateral (cis) dimers, and even tetramers [11]. EpCAM is probably mostly in a dimeric form at the cell surface, with only a small pool of monomeric protein [11].

The proposed function as homophilic CAM was based in particular on experiments using mouse L fibroblast (called L cells), which lack cadherins and show very low basal cell–cell adhesion. Ectopic EpCAM expression was sufficient to drive the formation of cell aggregates [8]. Note that these aggregates were significantly looser than those formed by L cells expressing classical E-cadherin. The same team detected a direct interaction between EpCAM cytoplasmic tail and the cytoskeletal cross-linker α-actinin, supporting the idea that EpCAM may be anchored to the actin cytoskeleton [19]. However, the actual adhesive function of EpCAM was already questioned early on by Litvinov et al. [20], who found that EpCAM expression decreased adhesion in E-cadherin-expressing cells. This result remains puzzling, since, as we will see below, EpCAM has a clear pro-adhesive activity, at least in the context of early embryos. As for the putative homotypic binding, however, recent extensive biochemical tests have failed to detect any hint of such interaction, and it was thus concluded that EpCAM is unlikely to be a bona fide CAM [21], reviewed in [13].

In the meantime, EpCAM was found to associate with claudin 7, a member of the claudin family, which are core transmembrane components of the tight junctions [22,23,24]. This interaction does not seem to occur at the level of the tight junctions, but rather involves a second pool of claudins distributed along the lateral side of epithelial cells, which is also precisely the site where EpCAM localises [11]. The EpCAM–claudin 7 interaction is essential for both proteins: It is responsible for their recruitment to TEMs [22,23] and for their stability [24,25]. In the absence of one of the two partners, the other is internalized and sent for lysosomal degradation [24,25]. Furthermore, the EpCAM–claudin association plays a role in the balanced distribution of claudin between the lateral pool and the tight junctional pool [24,26], although the impact of EpCAM on tight junction function is unclear: EpCAM depletion was found to have either no effect [26], or a slight positive effect [24], or, on the contrary, a deleterious effect on epithelial barrier integrity [27]. The latter observation was made under knock-out conditions [27], thus possibly resulting from a long-term absence of EpCAM. In the latter case, the phenotype may be worsened due to secondary effects such as inflammation resulting from the original developmental or metabolic defects.

## 3. EpCAM Function in Cell Signalling and Proliferation

The first clear evidence of a function of EpCAM independent of adhesion was the discovery of a signalling function of the short cytoplasmic domain [28]. This topic has been recently reviewed [29], and will be here only briefly summarized. The signalling cascade uncovered by Gires and colleagues starts with the shedding of the extracellular domain of EpCAM, which in turn triggers regulated intracellular proteolysis (RIP), resulting in the release of the cytoplasmic peptide. This peptide can bind to the adaptor four-and-a-half LIM domain protein 2 (FHL2) and to β-catenin. The three-component complex can enter the nucleus and interact with TCF/Lef1 transcription factors, activating targets of the canonical Wnt pathway [28,30]. One of the major effects of this cascade is to promote cell proliferation (typical β-catenin/TCF targets include Myc and Cyclin D), thus providing EpCAM with an oncogenic function. The same mechanism of RIP and activation of Wnt-β-catenin signalling was verified for EpCAM2/Trop2 [31]. The initial shedding has been shown to occur either by the action of the metalloproteinase α-secretase/ADAM17, or by the aspartic-type protease β-secretase/BACE1. ADAM17 is a classical extracellular metalloprotease, but high expression is restricted to selected cancer cell types [28]. BACE1 is widely expressed, but as other aspartic proteases, it is only active at an acidic pH. Thus, most of EpCAM proteolysis occurs in lysosomes [32]. Under these conditions, the cytoplasmic domain has little to no opportunity to activate the Wnt pathway, because it is also rapidly degraded [33]. It has been proposed that the tumour environment, which can be rather acidic, may allow extracellular shedding, and perhaps, under some particular conditions, more massive release of intact cytoplasmic domain and stimulation of Wnt signalling [32,34]. Other evidence for EpCAM signalling functions have been reported, including, for instance, activation of the EGF receptor and downstream Erk and Akt signalling by the shedded EpCAM extracellular domain [35]. In other systems, EpCAM expression was also found to negatively regulate Erk signalling [36]. The detailed mechanisms of these various signalling activities have not yet been defined. Altogether, these and other data have brought to light the capacity of EpCAM to influence in multiple ways intracellular signalling, transcriptional activity, cell proliferation and/or stemness as well as changes in genetic programs [37].

## 4. EpCAM Function in Tissue Morphogenesis

The central focus of this review covers a different role of EpCAM in modulating cell adhesion and migration. Most of our knowledge about this function does not come from cancer cells, but from the study of embryonic models.

### 4.1. EpCAM and Embryonic Development

The role of EpCAM during mammalian development remains ill-defined. EpCAM is widely expressed in the early mouse embryo, but becomes restricted to epithelial tissues and downregulated in the mesoderm at the time of gastrulation. Unfortunately, the expression of EpCAM2/Trop2 during early development has not been studied. EpCAM knock-out has yielded inconsistent phenotypes: In one case, it led to lethality due to defects in placenta development [38], while in two other cases, development was normal, except for defects in the intestine that led to rapid postnatal death [27,39]. Considering the strong phenotypes observed for EpCAM loss-of-function (LOF) described in Xenopus, the absence of early mouse phenotype is likely to be due to the presence of EpCAM2/Trop2, which presumably can compensate for the loss of EpCAM. Consistently, the intestine is apparently the only epithelial tissue that only expressed EpCAM and lacks EpCAM/Trop2, thus explaining the postnatal phenotype.

**Figure 1:**
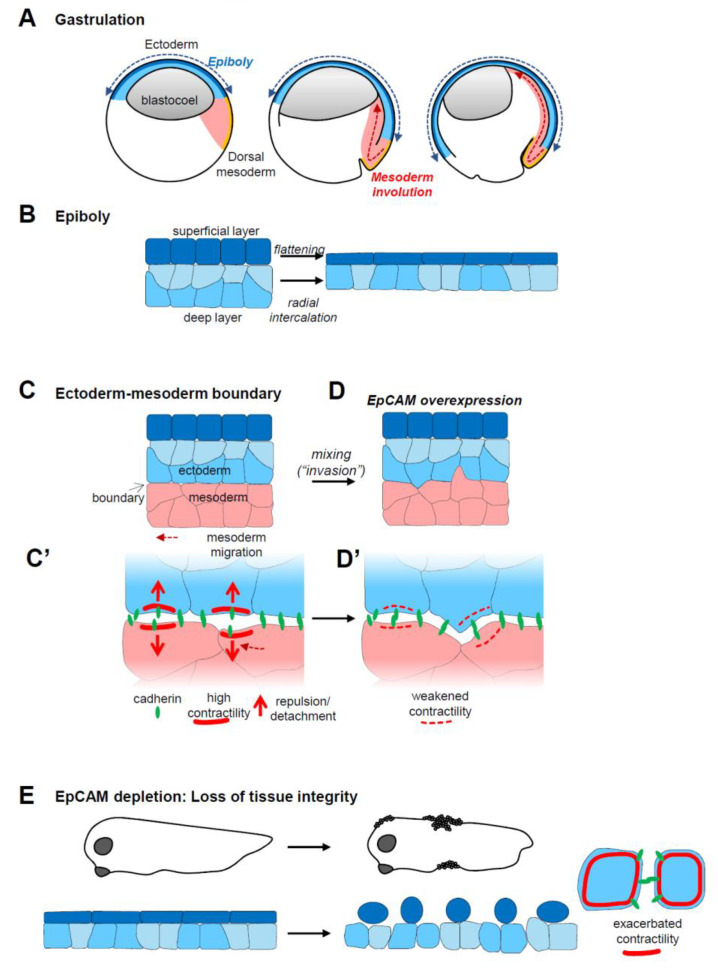
EpCAM gain-of-function and loss-of-functionembryonic phenotypes. (**A**) Diagram of three consecutive stages of Xenopus gastrulation, indicating the movement of ectoderm epiboly (blue) and of mesoderm involution (red). (**B**) Epiboly involves two morphogenetic movements: The cells of the superficial layer flatten, while the cells of the deep layer rearrange by radial intercalation to rearrange into a single layer. The combined action of these two movements results in a large expansion of the surface of the ectoderm, which, at the end of gastrulation, covers the entire embryo. (**C**) The ectoderm and mesoderm are kept separated by a sharp interface, a so-called embryonic boundary. The mesoderm migrates along the surface of the ectoderm, using ectoderm cells as substrate for adhesion. (**C’**) The boundary results from ephrin-Eph-mediated repulsive reactions that locally boost actomyosin contractility, which leads to local and transient detachments of cadherin adhesions across the boundary. Through alternate attachments and detachments, mesoderm migration can proceed without intermingling with the ectoderm. (**D**) High EpCAM expression decreases actomyosin contractility, perturbing the function of the boundary. This results in mixing between the ectoderm and mesoderm layers, blocking mesoderm involution. (**D’**) At the cellular level, reduced contractility abolishes repulsive reactions, favouring intimate adhesive contacts between the two tissues, and eventually leading to their intermingling. (**E**) EpCAM depletion leads to massive loss of tissue integrity, due to uncontrolled contractility that results in cells’ rounding up and disaggregation.

The study of EpCAM function in zebrafish and Xenopus embryos was more informative, as it revealed an important role in morphogenesis. Although, chronologically, data of fish were reported prior to those in Xenopus, I will start with the latter, as they are more complete and shed light on the zebrafish phenotypes. Xenopus EpCAM was originally identified by our team in a gain-of-function (GOF) screen for genes inducing mixing between ectoderm and mesoderm germ layers during gastrulation [40]. The screen was part of a project aimed at unravelling the process of tissue separation, using early segregation of the ectoderm and mesoderm germ layers as model. During gastrulation, the mesoderm migrates along the inner surface of the ectoderm (Figure 1A). For this purpose, mesoderm cells establish extensive cell–cell contacts with the ectoderm, using the latter as substrate for collective migration (Figure 1C). Despite this intimate relationship, the two tissues must maintain their integrity, which is provided by the formation of a so-called boundary. In the absence of this boundary, ectoderm and mesoderm cells start to intercalate, the two tissues fuse, mesoderm internalization stalls, and gastrulation aborts. The cellular mechanism responsible for maintenance of this boundary was recently identified (reviewed in [41,42,43]). It mainly relies on ephrin-Eph signalling across the tissue interface, which leads to bursts of local actomyosin contractility, temporarily destabilizing specifically ectoderm–mesoderm adhesive contacts. Note that at these early stages, EpCAM is not epithelial-specific, but ubiquitously expressed. Increasing its levels, either in the ectoderm or in the mesoderm, was sufficient to severely impair their separation (Figure 1D) [40].

EpCAM depletion did not affect tissue separation, but led to another gastrulation phenotype: It severely delayed epiboly, i.e., the morphogenetic process through which the ectoderm thins and spreads over the rest of the embryo (Figure 1B) [40]. Eventually, gastrulation was nevertheless completed. A few hours later, however, a second dramatic phenotype was observed [44]: Wounds started to appear at the surface of the embryo, from which dissociated inner cells spilled out. This turned out to reflect a generalized loss of tissue integrity (LEI) (Figure 1E). The embryonic tissues literally fell apart and the embryo rapidly died. We will see below that these apparently disparate phenotypes all derive from the same capacity of EpCAM to control myosin contractility.

Analysis of a zebrafish EpCAM LOF mutant showed strikingly similar phenotypes, i.e., a delay in epiboly and LEI [45]. However, the phenotypes were much less severe than in Xenopus. In particular, LEI was mild and only observed in the epidermis, while it affected the whole Xenopus embryo. This difference is likely due to the existence of two EpCAM genes in the tetraploid zebrafish: Only one of these genes was mutated/depleted in this study, and its loss was most likely compensated by the second EpCAM gene. In any case, the similarity with Xenopus argued for a conserved function. Note that EpCAM LOF showed additional phenotypes that were not further studied. In the post-gastrula Xenopus embryo, the notochord cells normally change shape and intercalate to adopt an organization resembling a stack of coins, a process that was blocked by EpCAM depletion (Maghzal and Fagotto, unpublished). The EpCAM mutant zebrafish showed defects in formation of the inner ear and of the lateral line [45,46]. The conservation of these phenotypes is not known, as EpCAM-depleted Xenopus embryos died before the appearance of these structures.

### 4.2. EpCAM Acts through PKC Signalling

In both zebrafish and Xenopus embryos, manipulation of EpCAM levels (both LOF and GOF) was followed by parallel changes in cadherin levels, indicating that EpCAM has a stabilization action on cadherins. The fish phenotypes were originally interpreted based on the assumed function of EpCAM as a cell adhesion molecule. EpCAM would thus cooperate with cadherins to reinforce cell–cell adhesive contacts [45]. However, our experiments in Xenopus unequivocally demonstrated that EpCAM embryonic function was independent of this putative CAM function. The key observation was obtained by comparing the action of full length EpCAM and a deletion construct lacking the whole extracellular domain (EpCAMΔE). Quite surprisingly, EpCAMΔEx was able to induce tissue mixing with the same efficiency as full length in GOF experiments, while another construct that included the extracellular domain but lacked the short intracellular tail (EpCAMΔC) was inactive [40]. Even more strikingly, EpCAMΔE could fully rescue the epiboly phenotype [40]. Note that EpCAMΔE could not rescue the later tissue integrity phenotype [44], an important observation that will be discussed below.

The activity of EpCAMΔE strongly argued for a signalling function. GOF and LOF experiments showed that EpCAM acted by inhibiting PKC kinases, more specifically members of the novel class of PKCs (nPKCs). Consistently, the EpCAM GOF tissue mixing phenotype was mimicked by treatment with specific nPKC inhibitors (but not inhibitors of classical PKCs) [40]. Furthermore, the epiboly phenotype caused by EpCAM depletion was fully rescued by nPKC inhibition [40]. Conversely, nPKC pharmacological activation was sufficient to mimic the epiboly defect [40]. nPKC inhibition also rescued LEI [44]. Thus, these major embryonic phenotypes could all be accounted for by the PKC-inhibitory property of EpCAM.

### 4.3. EpCAM Pro-Adhesive and Pro-Migratory Activity through Control of Myosin

**Figure 2:**
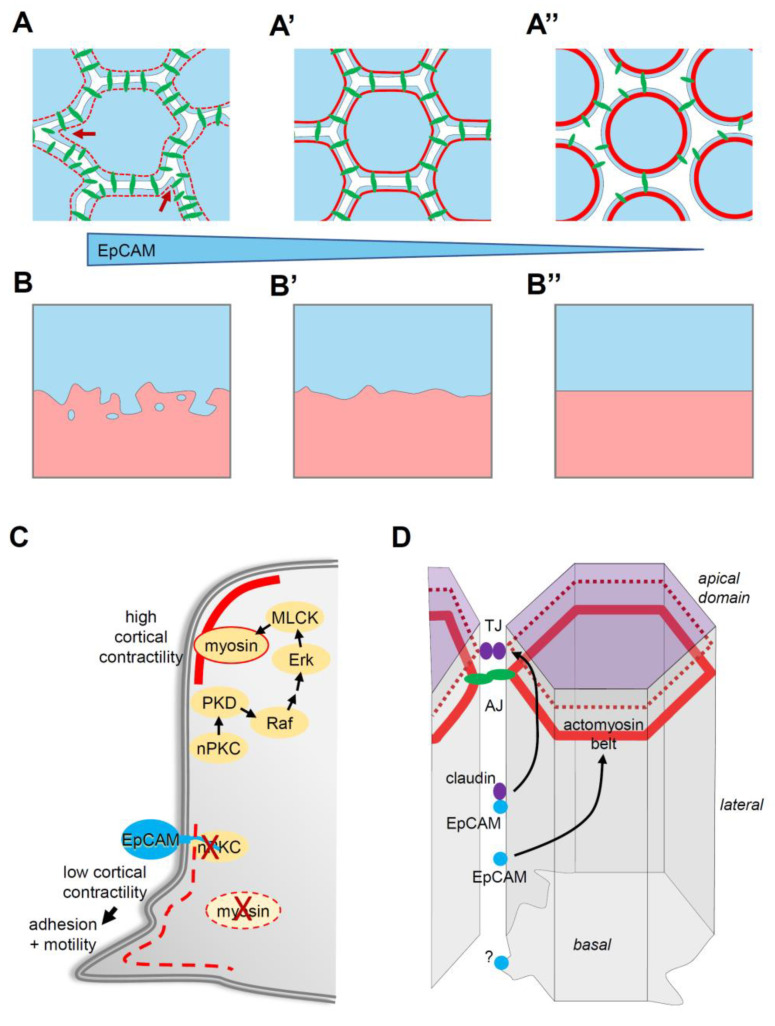
EpCAM, tissue plasticity and integrity. (**A**,**A’**) EpCAM levels regulate cell motility and adhesion: An increase in EpCAM levels (transition from (**A’**) to (**A**)) promotes intercellular migration by repressing actomyosin cortical contractility (from red continuous lines to dashed thin lines), which stimulates both protrusive activity (dark red arrows) and dynamic cell–cell adhesion (cadherins in green). (**A”**) Loss of EpCAM can lead to uncontrolled cortical contractility (thick red line), resulting in a strong decrease in or even loss of cell–cell adhesion. (**B**,**B’**,**B”**) EpCAM levels impact on tissue plasticity: The blue and pink surfaces represent two cell populations. At moderate levels (**B’**), intercellular migration is limited, and the two populations remain coherent. A high levels (**B**), cells actively migrate within the tissue and even between tissues, adopting an “invasive” behaviour, explaining in particular the embryonic tissue mixing phenotype. Low EpCAM-expressing cells (**B”**) fail altogether to migrate, due to impaired motility and adhesion. (**C**) Molecular mechanism responsible for control of myosin contractility. nPKCs activate one of the pathways that stimulates myosin activity and promotes high cortical contractility. This pathway involves the phosphorylation of PKD, a direct target of nPKCs, which triggers the Raf–Erk cascade. Erk can in turn activate myosin light chain kinase (MLCK). EpCAM cytoplasmic tail binds and inhibits nPKCs. This results in decreased myosin activity and cortical contractility, allowing protrusive activity and promoting cell adhesion. (**D**) In polarized epithelial cells, cadherin cell–cell adhesion is concentrated at adherens junctions (AJ), tightly associated with the so-called actomyosin belt. Proper AJ organization is required to establish and maintain functional tight junctions (TJ). Tight junctions also rely on association with an actin network (dark red dashed line). EpCAM is expressed along the lateral membrane. By inhibiting PKCs, it moderates actomyosin contractility. EpCAM also interacts with claudin, which are core components of the tight junctions. The EpCAM–claudin interaction may also participate in the regulation of tight junction organization and function. EpCAM has also been reported to interact with integrins, suggesting a potential role at the basal side in regulating cell–matrix adhesion and/or protrusive activity.

In both zebrafish and Xenopus, EpCAM manipulation positively influenced two prominent cellular parameters, cadherin levels and protrusive activity [40,44,45] (Figure 2A). Note that partial cadherin depletion potentiated tissue disaggregation in the EpCAM fish mutant, while cadherin overexpression rescued tissue integrity in EpCAM-depleted Xenopus embryos [44,45]. Nevertheless, the actual cause of the EpCAM phenotypes was not to be found in cadherin regulation, but rather in the capacity of EpCAM to repress myosin activity. Levels of phosphorylated myosin light chain, used as a read-out for myosin activation, were strongly decreased upon EpCAM overexpression, and increased upon its depletion, always in a PKC-dependent manner [40,44]. Functionally, the EpCAM GOF mixing phenotype could be rescued by experimental stimulation of myosin activity [40]. Conversely, development of EpCAM-depleted embryos could be fully rescued by simple treatment with the myosin inhibitor blebbistatin [44]. This capacity of EpCAM to repress myosin explained both its pro-migratory and pro-adhesive activities (Figure 2A,C): Protrusive activity was enhanced in cells expressing high EpCAM, while it was strongly reduced in EpCAM-depleted cells, consistent, respectively, with a weaker or, on the contrary, a more contractile actomyosin cortex [40]. The strength of cortical tension is also known to antagonize cell–cell adhesion. In the case of EpCAM depletion, exacerbated tension culminated with LEI [44]. All evidence supported the conclusion that stabilization of cadherin levels by EpCAM was an indirect effect downstream of myosin inhibition: High EpCAM levels stimulated adhesion by softening the cortex, thus allowing maximal engagement of cadherins, and, as a by-product, their stabilization at the cell contacts. Conversely, increased myosin activity of EpCAM-depleted cells tended to rigidify the cells, which impaired adhesion, secondarily leading to internalization and degradation of disengaged cadherins [44]. The combination of its pro-migratory and pro-adhesive activities provides EpCAM with the unique property of stimulating intercellular migration, i.e., migration of cells within a compact tissue (Figure 2A,B). We could show that by manipulating EpCAM levels in the ectoderm, we could experimentally control its properties, with low EpCAM levels yielding a highly coherent, non-motile configuration, while elevated levels would convert it into a dynamic tissue displaying an “invasive behaviour” [40] (Figure 2B). Note that this capacity to tune tissue plasticity only applies to “moderate” changes in EpCAM expression: On the one hand, EpCAM depletion eventually leads to LEI, thus trivially preventing any intercellular migration (Figure 2A”,B”), while, on the other hand, strong EpCAM overexpression also blocks migration. The reasons for the latter phenomenon remain to be determined. Among various hypotheses, I would mention the possibility that myosin activity may be reduced to levels too low to provide sufficient force for cell movement. Alternatively, another uncharacterized pathway may be affected under these conditions.

At any rate, myosin-dependent action on adhesion and migration could readily explain all the embryonic phenotypes: Cell intercalation of deep cells during epiboly is a typical case of the morphogenetic process based on intercellular migration, and, similarly, stretching of the superficial layer also involves remodelling the contacts both within this layer and with the underlying deep cells. EpCAM apparently contributes to achieving this plasticity by moderating ectoderm stiffness. Other gastrulation movements, i.e., mesoderm involution and endoderm vegetal rotation, do not seem to be sensitive to EpCAM depletion. Presumably, these tissues are soft enough [47,48,49] to maintain sufficient flexibility even in the absence of EpCAM. LEI, on the other hand, is an extreme phenotype, best explained by a self-feeding loop, where increased myosin contractility leads to contact destabilization and the loss of cadherins, which in turn further favours the stiffening of the cortex and deadhesion, until the cells eventually round up and dissociate.

### 4.4. The EpCAM-nPKC-Myosin Pathway

Molecularly, the repression of nPKC by EpCAM could not be more immediate. We could show that EpCAM directly binds nPKCs through a short juxtamembrane sequence. This sequence, which is highly conserved in all vertebrates, both for EpCAM and Trop2, acts as a pseudosubstrate. Pseudosubstrates are peptides that contain a sequence motif closely resembling the consensus sequences of enzyme substrates (here, nPKC substrates), but lack the target residue (here, a phosphorylatable serine or threonine). As such, these peptides act as efficient and specific inhibitors, by binding and masking the substrate recognition surface and preventing access to actual substrates. This turned out to be precisely the property of the EpCAM juxtamembrane domain [44]. Of note, PKCs are themselves autoinhibited by the binding of an internal pseudosubstrate sequence to the kinase domain. PKCs are typically activated by recruitment at the plasma membrane, where they are unfolded, thus relieving the autoinhibition. Whether EpCAM only binds nPKCs that already have an open active conformation, or whether they might even compete with the autoinhibitory internal pseudosubstrate, is not known.

Using LEI as a functional readout, we characterized the pathway connecting nPKC inhibition with myosin [44] (Figure 2C). The pathway involves PKCμ/PKD, a well-known direct target of nPKCs, which in turns activates the Raf–Erk pathway. Finally, Erk can stimulate myosin light chain phosphorylation by MLCK. There is a large number of potential substrates for the different components of this cascade, and multiple possible routes that could eventually input on myosin, and/or on other aspects of the actin cytoskeleton and of cell adhesion (see examples in Table 1). Yet, inhibition of the nPKC–PKD–Erk pathway was fully sufficient to account for the requirement for EpCAM in tissue integrity [44]. One hypothesis could be that EpCAM would target a particular pool of these signalling components, which might be mostly dedicated to control actomyosin cortical contractility, Intriguingly, however, our observations revealed rather global effects of EpCAM GOF and LOF on both PKC and Erk activity [40,44]. Alternatively, EpCAM may indeed modulate multiple targets downstream of nPKCs, but the myosin “branch” may be the most sensitive to EpCAM regulation, at least during embryonic development.

### 4.5. EpCAM Function in Intestinal Homeostasis

I already mentioned that, in mice, the EpCAM knock-out does not seem to affect embryonic development (except possibly for the placenta), but compromises the barrier function of the intestine. This function has been characterized in some detail in human and mouse intestinal cells, as it directly relates to a human disease, congenital tufting enteropathy (CTE), which is precisely characterized by LOF EpCAM mutations [50]. The barrier defect could obviously be connected to the EpCAM–claudin interaction, which was precisely the model proposed in two studies [25,27] (Figure 2D). However, the Delacour team came up with a different model, based on the ability of EpCAM to control myosin [51] (Figure 2D). Using intestinal biopsies from CTE patients and Caco2 intestinal cells depleted of EpCAM, they observed LEI, which they further characterized. They observed the loss of cadherin-based adherens junctions, enlarged the apical domain at the expanses of the lateral domain, and disorganized tight junctions [51]. The authors focused on the tricellular junctions, which, being the “vertex” of the junctional system, are expected to bear the highest tension. They demonstrated that the phenotype could be explained by elevated myosin activity. In particular, treatment with the myosin inhibitor blebbistatin could rescue epithelial integrity, exactly like in the case of the Xenopus embryo. Consistent with the EpCAM–myosin connection, we had previously showed that EpCAM depletion in Caco2 cells led to upregulation of PKC and Erk activity and elevated myosin phosphorylation [44]. A similar Erk–myosin inhibitory role was recently demonstrated in the renal epithelial MDCK line [26]. Thus, the role of EpCAM in the integrity of differentiated epithelia appears to be identical to the one identified in the early Xenopus embryos. The lack of reports on similar effects in other epithelial tissues and cell lines is likely due to the frequent coexpression of EpCAM and EpCAM2/Trop2. The redundancy of the two genes was recently demonstrated in keratinocytes [52].

## 5. Perspectives: From Here, Now Where Do We Go?

EpCAM appears to play a major role in controlling tissue properties. By tampering myosin, it stimulates both motility and cell–cell adhesion, a combination ideally suited to drive intercellular migration within a coherent tissue. One may qualify this state as “plastic” (Figure 2). While this discovery opens exciting perspectives about EpCAM function during development and in cancer, we still are at the very beginning of exploring this aspect of EpCAM biology. I mention here a few major questions that need to be addressed.

### 5.1. Is EpCAM Specifically Controlling Cortical Tension?

The ability of EpCAM to promote, at the same time, adhesion and migration solely through the inhibition of myosin is quite remarkable. The role of myosin in building tension of the cell cortex is certainly essential, and relaxing this tension is a prerequisite for cell spreading, the formation of cell protrusions and the establishment of adhesive contacts. However, myosin also has an essential pro-adhesive function in building the contractile fibres that anchor adhesive junctions. This mechanical coupling of adhesive structures has, in turn, a complex relationship with migration: Adhesion is necessary for migration, as cells need to grab a substrate or another cell to move, but at the same time migration also requires remodelling of adhesive contacts. Thus, by strengthening and stabilizing adhesions, myosin activity can also slow down or ultimately even stall migration. This two-fold duality of myosin function, i.e., cortical tension versus anchoring, and stable adhesion versus dynamic migration, accounts for the wide range of adhesive and migratory phenotypes that can be observed upon experimental manipulation of myosin activity, and explains the numerous contradictions found in the literature. However, the fact remains that the EpCAM GOF and LOF data from embryonic tissues and intestinal cells are surprisingly coherent, arguing that EpCAM primarily controls cortical tension. How EpCAM targets this specific myosin function, and how it manages in setting the right balance of tension required for proper tissue plasticity is a key and currently widely open question. The only piece of information that we have gathered so far was that EpCAMΔE could not rescue LEI, although it retained the capacity to downregulate PKC and myosin [44]. This is consistent with the hypothesis that EpCAM chiefly controls local activities at the cell periphery, perhaps even in subdomains of the plasma membrane, probably in association with other regulatory components.

### 5.2. How Much Specificity Can Be Achieved by Controlling Multifunctional Components?

The apparent specificity of EpCAM function is even more stunning when one considers that it involves a cascade constituted of kinases with pleiotropic functions. The apparent global impact of EpCAM GOF and LOF on PKC and Erk activities is difficult to reconcile with the specific cell and tissue phenotypes, all related to myosin regulation.

The beginning of an answer may be found in the capacity of all these signalling components to associate into specialized complexes, which restrict their action to specific cellular compartments. Focal adhesions are the best characterized example of structures assembling specific signalling complexes, also including Raf, MEK and Erk [53]. While these complexes are best known in the context of the crosstalk between adhesive structures and the nucleus, clearly much signalling activity is concentrated at the cell periphery, and must thus primarily act on local targets. Consistently, live imaging has revealed strong and sustained Erk activation at the plasma membrane, contrasting with transient activation in the nucleus [54]. Similarly, PKD also interacts with a variety of components, including several actin regulators (reviewed in [55]). It may not be far-fetched to postulate the existence of a Raf–Erk module centred on MLCK and myosin, yet to be characterized.

Note that the fact that EpCAM phenotypes could be fully rescued by manipulating Raf or Erk, while providing the demonstration of the importance of this pathway, did not exclude the possibility that EpCAM may also regulate additional routes, which could also converge on myosin and/or other components of the actin cytoskeleton. Interestingly, nPKC–PKD signalling is not as promiscuous as it may seem. PKD is not an indiscriminate target of all PKCs, but is clearly preferentially activated by the novel class of PKCs [55]. Furthermore, a large number of nPKCs and PKD substrates are directly related to the actin cytoskeleton and to adhesive structures. I have compiled examples in Table 1, which include important components, such as Src, β-catenin, cofilin and its upstream regulators, various GAPs and GEFs, only to mention a few. Identifying relevant targets controlled by EpCAM with potential impact on morphogenetic properties is a fully open avenue that is certainly worth pursuing. Conceivably, EpCAM might selectively control a set of modules specialized in cytoskeleton regulation.

### 5.3. Regulation

One other important issue for which one currently lacks experimental evidence concerns the potential regulation of EpCAM. The scenario of a plainly constitutive nPKC inhibition without other control than EpCAM levels seems unlikely, and it seems reasonable to hypothesize the existence of regulatory mechanisms, involving for instance sequestration to membrane subdomains, protein–protein interactions, or EpCAM stability at the membrane. I already mentioned the requirement for the extracellular domain, which could reflect a role of EpCAM dimerization and/or association with other partners. The interaction with claudin 7 (and with TEMs) is an important lead to pursue, as it could impact not only on their mutual stability [24,25], but also on subcellular localization and/or on PKC inhibition. EpCAM has also been detected as an interactor of integrins [18,56,57,58] (Figure 2D), but the physiological implications are so far unclear.

The position of the inhibitory pseudosubstrate sequence, adjacent to the lipid bilayer, appears optimal for the efficient inhibition of membrane-bound PKC [44]. Thus, the release of the EpCAM in the cytoplasm by RIP [28,29,30,31] would be predicted to abolish EpCAM inhibitory function. Another potential protease-dependent regulatory mechanism involves cleavage of the extracellular domain by an extracellular protease called matriptase [52,59]. In this case, the small N-terminal product remains connected to the rest of the protein through disulphide bridges, but the properties of EpCAM are affected, including stability at the plasma membrane [52,59], but also cis-dimerization [60] and lateral interactions with claudins [59], all events that could impact on EpCAM function.

### 5.4. EpCAM Morphogenetic Function and Cancer

EpCAM’s ability to stimulate tissue plasticity is likely to be an important facet of the role of EpCAM in cancer, and the next major challenge will be to build on the knowledge obtained from embryonic tissue to define the morphogenetic potential of EpCAM during cancer development and metastasis. Yet, it is currently difficult to make strong a priori predictions of whether EpCAM may act as a pro-invasive factor, or, on the contrary, as a repressor of invasion. Even assuming that the role of EpCAM is solely restricted to moderating cortical tension, it may have different effects on invasion, depending on the type of cancer and on the context. The most tempting hypothesis is that EpCAM, by increasing the dynamicity of tissues, may favour collective invasion. However, it is also possible that under some conditions, stimulation of motility may dominate over cell–cell adhesion, in which case EpCAM might help single cells to escape from the tumour. On the contrary, it could increase tissue cohesion, thus acting as suppressor of invasion. One interesting aspect of tumorigenesis that is usually eluded is the need of intercellular migration within the growing tumour. Indeed, continuous proliferation within a compact mass necessarily implies that cells must be able to rearrange. In this context, tumour rigidity could become a limiting factor, which could be alleviated by EpCAM’s “plasticizing” action.

At least superficially, the fact that EpCAM-mediated inhibition of the PKC–PKD–Raf–Erk cascade favours migration, and thus potentially invasion, is at odds with the fact that all the components of this cascade are classically viewed as oncogenes, well-known for their implication in cell proliferation, inhibition of apoptosis and tumorigenesis. Erk is also typically considered as an activator of migration and invasion [61]. However, it is now clear that all these regulators can act both as oncogenes or tumour suppressors, and, in terms of invasion, they can either stimulate or repress migration (e.g., [61,62,63]). The intrinsic complexity of these pathways, their involvement at multiple stages of tumour growth and cancer dissemination raises scepticism about the efficiency of using kinase inhibitors as anti-cancer drugs. If the hypothesis of distinct complexes dedicated to specific tasks holds true, a more rational approach would be to focus on the identification of key protein–protein interactions involved in recruiting these kinases to distinct modules. One may then be able to more selectively perturb one of their functions while shifting the balance toward an antagonistic process. Thus, assuming the existence of a PKD–Raf–Erk module dedicated to myosin, the identification of potential distinct complexes would be a huge step that could ultimately allow the design of refined drugs. As for EpCAM, the fact that its cleavage, dimerization, interaction with claudins and PKC inhibition may all be interdependent reactions may offer, in the future, a great opportunity to design specific tools to manipulate this morphogenetic pathway. There is still yet a long way to go, and further in-depth exploration will no doubt reveal new unforeseen properties of this fascinating molecule.

## Figures and Tables

**Table 1:** nPKCs and PKD substrates related to the cytoskeleton and adhesive structures.

Gene	Full Name/Alternate Name	Functions/Comments	Kinase
**Cytoskeleton**			
ADD1	adducin 1	assembly of spectrin–actin network	PKCδ
ARHGAP3	β2 chimaerin	RacGAP	PKCδ
Arhgef15	ephexin-5	RhoGEF	PKCε
CENTA1	ADAP1	ArfGAP	PKCε
CFL1	cofilin 1	actin turnover	PKD
CORO1B	coronin 1B		PKCε
CTTN	cortactin	actin organization	PKCδ, PKD
DLC1	deleted in liver cancer 1	RhoGAP	PKD
GIT1		ArfGAP, adhesion and migration	PKD
HAX1	HCLS1-associated protein X-1	regulates Arp2/3 recruitment to cortex	PKCδ
IQGAP1		binds activated CDC42, scaffold protein	PKCε
LCP1	L-plastin	actin-binding protein	PKCδ
LIMK2	LIM kinase 2		PKCδ
MYPC3	myosin-binding protein C		PKCδ
MARK2	Ser/Thr-protein kinase	cell polarity, microtubule dynamics	PKD
MIIP	migration/invasion-inhib prot		PKCε
PAK4	p21-activated kinase 4	activated by cdc42 and Rac1	PKCδ, PKD
PIP5K1B	PIP5 kinase 1β	Rac1-dep. reorganization actin filaments	PKCδ
PLCB3	phospholipase C-β-3		PKCε
PLD2	phospholipase D2	signal-induced cytoskeletal regulation	PKCδ
Plekhg5		RhoGEF	PKD
PPP1R14A,B	PP1 regulatory subunit14A,B	myosin regulation	PKCδ,ε,PKD
PREX1	RacGEF	Rac activator	PKCδ
PRKD	***PKD***		PKCδ,ε
RASGRP3	GEF for Ras and Rap1		PKCδ
REM1		actin cytoskeletal reorganization	PKD
Rhotekin		Rho effector	PKD
Src	Src kinase		PKCδ
SHH3	phosphatase Slingshot homolog 1	cofilin activation	PKD
TAGLN	Transgelin	actin cross-linking/gelling protein	PKCδ
VASP		actin nucleator	PKD
**Cell–Cell and Cell–Matrix Adhesion**			
CDH2	N-cadherin		PKD
CIB1	calcium and integrin-binding protein 1		PKD
CTNNB1	β-catenin		PKCδ,ε,PKD
ITGB1	Integrin β1		PKCη
ITGB2	Integrin β2		PKCδ,ε
ITGB4	Integrin β4		PKD
PTPRA	recept tyr phosphatase α	integrin–Src–PAK–Rac signalling	PKCδ
PXN	paxillin	major integrin–actin cross-linker	PKCδ
SDC4	syndecan-4	cell surface proteoglycan/binds fibronectin	PKCδ
**Tight Junctions**			
OCLN	occludin	Core component	PKCε
Tjp1,2	ZO1,2	adaptor, linker to actin, signalling	PKCε

The list of validated nPKC and PKD substrates was selected from the PhosphoSitePlus database, based on known activities impinging on the actin cytoskeleton and/or on adherens junctions and tight junctions.
